# Computational holographic Maxwellian near-eye display with an expanded eyebox

**DOI:** 10.1038/s41598-019-55346-w

**Published:** 2019-12-10

**Authors:** Chenliang Chang, Wei Cui, Jongchan Park, Liang Gao

**Affiliations:** 1 Department of Electrical and Computer Engineering, University of Illinois at Urbana-Champaign, 306 N. Wright St, Urbana, 61801 IL USA; 2 Beckman Institute for Advanced Science and Technology, University of Illinois at Urbana-Champaign, 405 N. Mathews Ave, Urbana, 61801 IL USA

**Keywords:** Displays, Imaging and sensing

## Abstract

The Maxwellian near-eye displays have attracted growing interest in various applications. By using a confined pupil, a Maxwellian display presents an all-in-focus image to the viewer where the image formed on the retina is independent of the optical power of the eye. Despite being a promising technique, current Maxwellian near-eye displays suffer from various limitations such as a small eyebox, a bulky setup and a high cost. To overcome these drawbacks, we present a holographic Maxwellian near-eye display based on computational imaging. By encoding a complex wavefront into amplitude-only signals, we can readily display the computed histogram on a widely-accessible device such as a liquid-crystal or digital light processing display, creating an all-in-focus virtual image augmented on the real-world objects. Additionally, to expand the eyebox, we multiplex the hologram with multiple off-axis plane waves, duplicating the pupils into an array. The resultant method features a compact form factor because it requires only one active electronic component, lending credence to its wearable applications.

## Introduction

Compact and portable, near-eye displays hold great promises in augmented reality (AR), virtual reality (VR) and mixed reality (MR) applications such as social communication, healthcare, education, and entertainment^1,2^. In the past decade, various optical schemes have been adopted in near-eye 3D displays. Representative implementations encompass light field displays^3–5^, freeform surfaces^6,7^, multi-focal-plane displays^8,9^, and holographic related display techniques including holographic waveguide^10–12^ and holographic optical element (HOE)^13–15^. Capable of providing depth cues that mimic the real world, these methods reduce the eye fatigue by alleviating the vergence-accomodation conflict (VAC)^16^. However, solving one problem creates another—because of complicated optics employed, current near-eye 3D displays generally suffer from a large form factor^9^ and/or a low resolution^5^, limiting their use in wearable devices.

**Figure 1:**
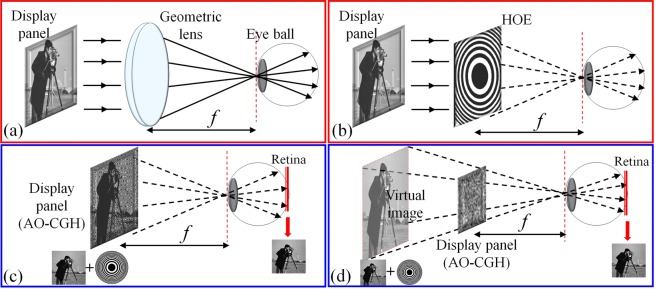
Operating principle. (**a**) Maxwellian display using a geometric lens. (**b**) Maxwellian display using a HOE. (**c**) Holographic Maxwellian display based on direct complex wavefront encoding. (**d**) Holographic Maxwellian display based on computational complex hologram encoding. (The schematic image of “cameraman” in this figure is a popularly used standard test image that can be downloaded online from one of the test images database http://www.imageprocessingplace.com/root_files_V3/image_databases.htm).

As an alternative approach, a Maxwellian display solves VAC while still maintaining a high resolution^17^ in a compact enclosure. In an imaging system, the beam width limited by the pupil diameter determines the depth of field (DOF) within which the image is considered in focus^18^. In Maxwellian displays, the chief rays emitted from the display panel converge at the eye pupil and then form an image at the retina (Fig. 1a). By using a narrow pencil beam, Maxwellian displays reduce the effective pupil size and thereby significantly increase the DOF—the image appears in focus at all depths. The ability of creating this all-in-focus image thus provides a simple solution to eliminate the VAC because the eye lens does not need to accommodate the virtual object.

Despite being a promising technique, Maxwellian displays have a major drawback in the eyebox size because of the small pupil employed. To expand the eyebox, there are generally two strategies: temporal multiplexing and spatial multiplexing. The temporal-multiplexing-based Maxwellian displays rapidly translate the pupil by using either a LED array^19^ or dynamic tilted mirrors^20^. Because the scanning must be performed within the flicker-fusion threshold time, the temporal-multiplexing-based Maxwellian displays face a fundamental trade-off between the image refreshing rate and eye box size. By contrast, the spatial-multiplexing-based Maxwellian displays^21,22^ use HOEs to simultaneously create multiple laterally-shifted, duplicated pupils, expanding the eyebox while maintaining the original frame rate. Additionally, because HOEs can be directly fabricated on waveguides, the resultant devices normally feature a compact form factor. Nonetheless, the fabrication of HOEs is nontrivial—the recording interference patterns from two coherent beams requires extensive alignment and stable optical setups. More problematically, the HOEs cannot be modified once they are fabricated. Alternatively, the spatial multiplexing can also be achieved through computer-generated holography (CGH)^23^, which computes and displays a histogram on an electronic device like a spatial light modulator. Despite being flexible in the hologram created, the current CGH implementations require additional eye pupil tracking devices^24^ or HOEs^23^, resulting in a bulky and complicated setup.

To overcome these limitations, herein we present a computational holographic Maxwellian near-eye display. After multiplexing different directional plane carrier waves to the virtual target image, we first compute a multiplexing complex hologram based on free-space Fresnel diffraction. This hologram converges the light rays into multiple pupils and produce Maxwellian all-in-focus images at each pupil location. Next, we encode this complex hologram into an amplitude image to facilitate its display on a commonly-accessible device such as a liquid-crystal display (LCD) or a digital light processing (DLP) display. Our method enables high-resolution Maxwellian display with an expanded eyebox in a compact configuration.

## Principle and Method

We illustrate the operating principle of the Maxwellian near-eye display using a geometric lens and HOE (i.e., a diffractive lens) in Fig. 1(a,b), respectively. Under monochromatic illumination, the complex light wavefront right after the lens plane is *A·*exp(i*φ*), where *A* is the amplitude and *φ* is the phase. Herein the display panel produces the image amplitude *A*, and the geometric or diffractive lens generates the converging spherical phase *φ*. This complex wavefront propagates and converges to a focal spot at the pupil plane. Therefore, the key to create a holographic Maxwellian view is to modulate the wavefront in a way that combines the amplitude function of a target image and the phase function of a lens.

Figure 1(c,d) show two basic configurations of holographic Maxwellian near-eye displays, respectively. In Fig. 1(c), the complex hologram is a superimposed image of a real target image and a spherical phase. The light emitted from this hologram enters the eye pupil and forms an image on the retina. Because the target image is close to the eye (the focal length *f* is usually small in near-eye displays), the DOF is relatively small^24^. By contrast, in Fig. 1(d), the target image is virtual, and it locates at a far distance from the eye pupil. To compute the hologram, one need to forward propagate the wavefront from the virtual target image to the display panel. Because it yields a larger DOF than that presented in Fig. 1(c) ^24^, we adopt this method in experiments. Next, we encode this complex amplitude into an amplitude-only CGH (AO-CGH) using a holographic complex beam shaping technique^25,26^. The resultant AO-CGH can be displayed on a conventional micro-display device and provide a Maxwellian-view image. Because only a display panel is required, our method is simple and low cost. Moreover, it can be readily combined with various optical combiners such as a waveguide.

### Calculation of complex holograms

**Figure 2:**
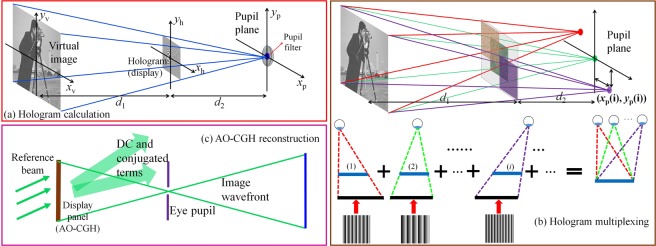
Computation and reconstruction of holograms. **(a**) Geometry model for computation. (**b**) Hologram multiplexing procedure. (**c**) Geometry model for reconstruction. (The schematic image of “cameraman” in this figure is a popularly used standard test image that can be downloaded online from one of the test images database http://www.imageprocessingplace.com/root_files_V3/image_databases.htm).

We illustrate the basic model for calculating the complex hologram in Fig. 2(a). The system consists of a virtual image plane, a display plane, and an eye pupil plane. The virtual target image is compulsively multiplied by a spherical converging phase factor. This operation is equivalent to illuminating the image by virtual converging light rays which focus at the eye pupil. Therefore, the wavefront at the virtual image plane can be written as

1 $$V({x}_{v},{y}_{v})=I({x}_{v},{y}_{v})\cdot \exp [\frac{-ik({x}_{v}^{2}+{y}_{v}^{2})}{2({d}_{1}+{d}_{2})}],$$

where *I*(*x*_v_, *y*_v_) is the amplitude of the target image, *k* = 2π/*λ* is the wave number. *d*_1_ and *d*_2_ are the distances from the virtual image to the display plane and from the display plane to the eye pupil plane, respectively. The complex hologram at the display plane can be calculated from the virtual image using the forward Fresnel diffraction approximation^27^ as

2 $$H({x}_{h},{y}_{h})=\iint V({x}_{v},{y}_{v})\cdot \exp \{\frac{ik}{2{d}_{1}}[{({x}_{h}-{x}_{v})}^{2}+{({y}_{h}-{y}_{v})}^{2}]\}d{x}_{v}d{y}_{v}.$$

Here *H*(*x*_h_, *y*_h_) denotes the complex-amplitude distribution of the hologram. Equation (2) can be numerically calculated by employing the “ARSS-Fresnel diffraction” algorithm^28,29^ which involves convolution using three fast Fourier transforms (FFTs). The resultant complex hologram can yield an sharp image on the retina plane through eye pupil filtering of the focal spot, visually equivalent to staring at a virtual image whose distance is *d*_virtual_ = *d*_1_ + *d*_2_ in front of the eye.

### Eyebox expansion through pupil duplication

To expand the eyebox, we further calculate a multiplexing complex hologram to generate multiple laterally shifted pupils at the eye pupil plane. As illustrated in Fig. 2(b), we first calculate multiple complex sub-holograms at the display plane using Fresnel diffraction in Eq. 2. Each sub-hologram is associated with a duplicated pupil, and we calculate it by applying an individual plane carrier wave (blazing tilting phase) at angle (*θ*_x(i)_, *θ*_y(i)_) to the virtual target image *V*(*x*_v_, *y*_v_). Here *i* denotes the sub-hologram index and the *i*-th sub-hologram is therefore calculated by:

3 $${H}_{i}({x}_{h},{y}_{h})=\iint V({x}_{v},{y}_{v})\cdot \exp [ik(\frac{{x}_{v}{\theta }_{x(i)}}{({d}_{1}+{d}_{2})}+\frac{{y}_{v}{\theta }_{y(i)}}{({d}_{1}+{d}_{2})})]\cdot \exp \{\frac{ik}{2{d}_{1}}[{({x}_{h}-{x}_{v})}^{2}+{({y}_{h}-{y}_{v})}^{2}]\}d{x}_{v}d{y}_{v}.$$

Finally, we multiplex all sub-holograms into a composite hologram:

4 $${H}_{c}({x}_{h},{y}_{h})=\mathop{\sum }\limits_{i=1}^{n}{H}_{i}({x}_{h},{y}_{h}).$$

Adding different plane carriers to the virtual image allows independently steering the beam toward given directions. This operation enables computational modulation of multiple beams, converging them at different pupils simultaneously. We calculate the angles for the *i*-th pupil as *θ*_x(i)_ = *x*_p_(i)/(*d*_1_ + *d*_2_), *θ*_y(i)_ = *y*_p_(i)/(*d*_1_ + *d*_2_), where (*x*_p_(i), *y*_p_(i)) denotes the center coordinate of the *i*-th pupil. The distance between adjacent pupils (*x*_p_(i + 1)-*x*_p_(i), *y*_p_(i + 1)-*y*_p_(i)) is larger than the eye pupil size so the eye sees a single pupil at a time when it moves, thereby expanding the effective eyebox.

To display the complex hologram on the amplitude displays, we encode the complex amplitude given by Eq. (4) into an AO-CGH. Given a complex amplitude *H*_c_(*x*_h_, *y*_h_) = *a*(*x*_h_, *y*_h_)·exp[*iφ*(*x*_h_, *y*_h_)], where the amplitude *a*(*x*_h_, *y*_h_) is a positive normalized function and the phase *φ*(*x*_h_, *y*_h_) takes values in the domain [−π, π], we can encode it into an interferometric AO-CGH with a normalized transmittance function

5 $$A({x}_{h},{y}_{h})={c}_{0}\{b({x}_{h},{y}_{h})+a({x}_{h},{y}_{h})\cdot \,\cos [\varphi ({x}_{h},{y}_{h})-2\pi ({u}_{0}{x}_{h}+{v}_{0}{y}_{h})]\},$$

where *c*_0 _≅ 1/2 is a normalization constant, (*u*_0_*, v*_0_) are the spatial frequencies of the linear phase hologram carrier, and *b*(*x*_h_, *y*_h_) is the bias function generally defined as *b*(*x*_h_, *y*_h_) = [1 + *a*^2^(*x*_h_, *y*_h_)]/2. Generation of the hologram transmittance in Eq. (5) is optically equivalent to recording the interference pattern formed by the complex hologram *H*_c_(*x*_h_, *y*_h_) with an off-axis plane reference wave exp[*i*2π(*u*_0_*x*_h_ + *v*_0_*y*_h_)]. The main spectral band of the encoded AO-CGH only presents the off-axis signal term (i.e., the desired complex amplitude to generate the pupil array) accompanied by the DC term and the conjugate of signal. The isolation of the off-axis signals from the DC and conjugate can be achieved through pupil filtering, as shown in Fig. 2(c), enabling high-quality image reconstruction on the retina. It is noteworthy the parameterization of Eq. 5 is not unique. For instance, rather than using its general definition, we can choose the bias *b*(*x*_h_, *y*_h_) in Eq. (5) in other forms to make the transmittance *A*(*x*_h_, *y*_h_) positive^26^.

It should be emphasized that in conventional Maxwellian display, the electronic screen displays the image amplitude while the refraction lens (or diffractive lens such as HOEs) alters the phase of the light. By contrast, in our method, we encode both the image amplitude and phase into a single hologram using wavefront modulation, eliminating the need for the focusing lens and thereby leading to a more compact form factor. Moreover, because the image is reconstructed by wavefront modulation, we can correct for the aberrations of the system simply by digitally adding an auxiliary phase to the wavefront, thereby offering more flexibilities in improving the image quality^24^.

## Experiments and Results

**Figure 3:**
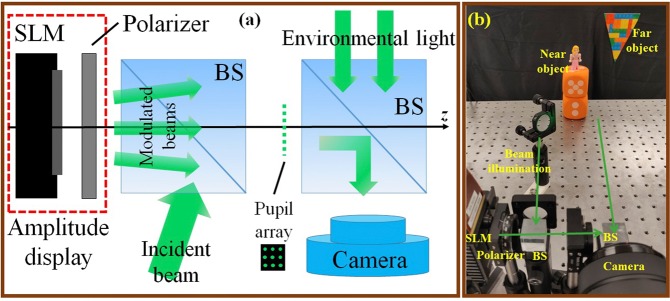
Optical setup. (**a**) System schematic. (**b**) Photograph.

To demonstrate our method, we built a prototype using only off-the-shelf optics. The system schematic and photograph are shown in Fig. 3(a,b), respectively. Our amplitude display consists of a phase spatial light modulator (SLM) (Meadowlark, 9.2 μm pixel pitch, 1920 × 1152 resolution) and a linear polarizer oriented 45° with respect to the *x*-axis^30^. We load AO-CGH with a 1024 × 1024 resolution into SLM, and we obliquely illuminate the SLM with a 532 *nm* laser beam. To characterize the image quality seen from each pupil, we translated an iris at the pupil plane. The resultant image is then captured by a digital camera which consists of a CMOS sensor (Sony Alpha a7s) and a varifocal lens (focal distance: 450 *mm* to infinity).

**Figure 4:**
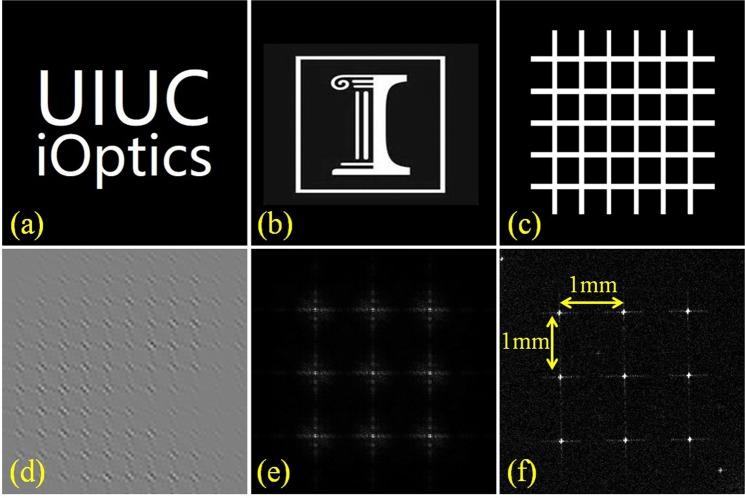
Test target images and pupil array. (**a**–**c**) Target images. (**d**) Representative AO-CGH. (**e**) Simulated pupil array. (**f**) Experimentally-measured pupil array.

We used three test images (Fig. 4a–c) in our proof-of-concept experiments. To calculate the correspondent hologram, we set *d*_1_ = 350 *mm*, *d*_2_ = 150 *mm*, so the resultant virtual image is located at *d*_virtual_ = 350 *mm* + 150 *mm* = 500 *mm* in front of the eye. Figure 4(d) shows a representative AO-CGH associated with Fig. 4(a). The composite AO-CGH for eyebox expansion is calculated by multiplexing nine sub-holograms with different plane carrier waves, yielding a 3 × 3 pupil array at the eye pupil plane. The distance between adjacent pupils is 1 *mm* along both axes. By placing a monochromatic CCD (PointeGray Chameleon3) directly at the pupil plane, we captured the pupil array image (Fig. 4(f)), which closely matches with simulation (Fig. 4(e)). In our experiments, the distance between adjacent pupils is limited by the SLM pixel size. We could increase this separation by using a SLM with a smaller pixel pitch and thereby a larger diffraction angle.

**Figure 5:**
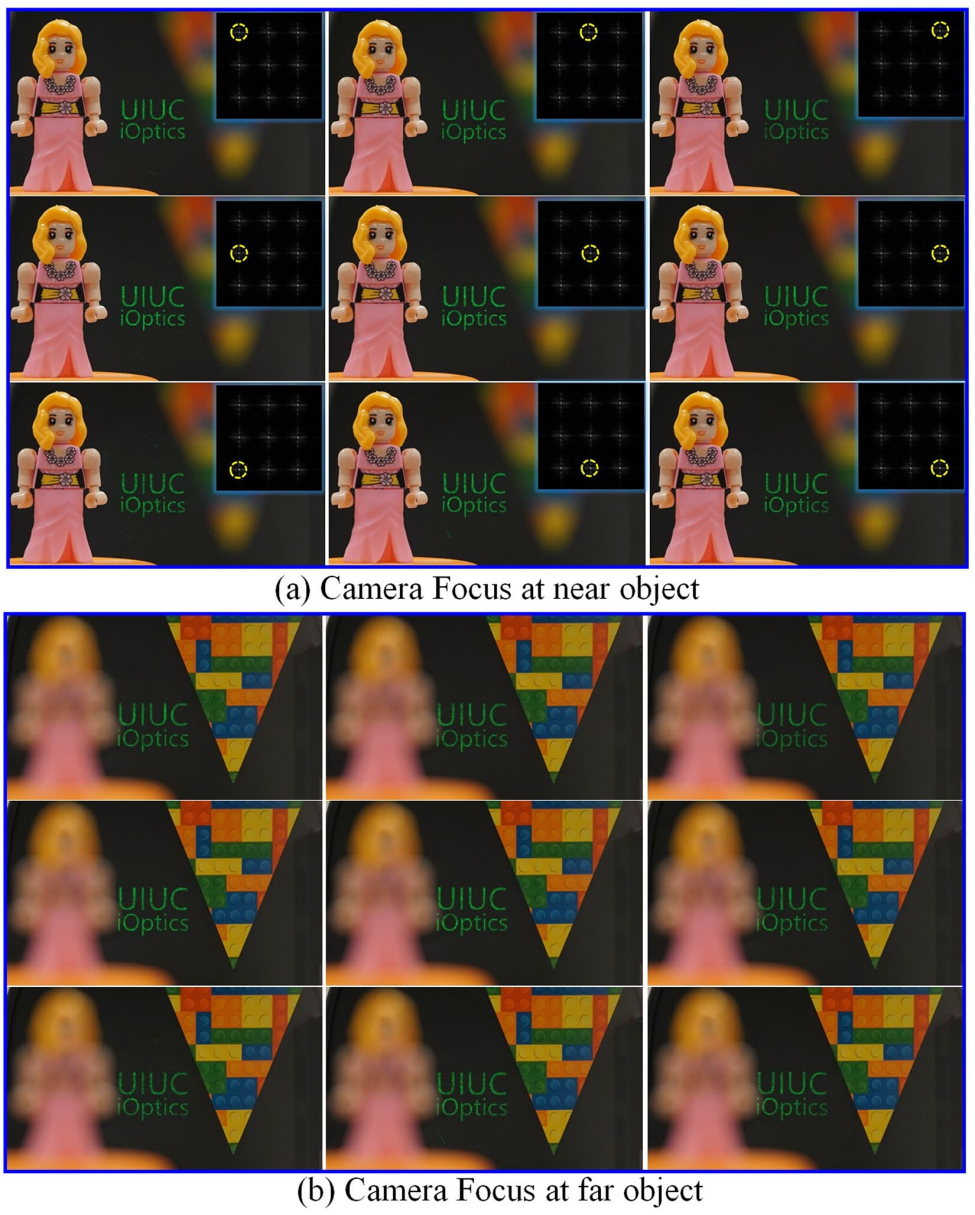
Images of a test target (“letters”) seen through different pupils at varied focal distances. (**a**) Focus at a near object (2 diopters). (**b**) Focus at a far object (0.4 diopters).

We moved the iris to nine pupil locations. Figure 5 shows the captured images by the camera behind the iris, and in the insets we labeled the correspondent iris location at the eye pupil plane. Because of using an optical combiner, we see both the reconstructed image and real objects. We varied the focal distance of the camera from 2 diopters to 0.4 diopters to focus on a near and far real-world object, respectively, while the reconstructed virtual image is always in focus.

**Figure 6:**
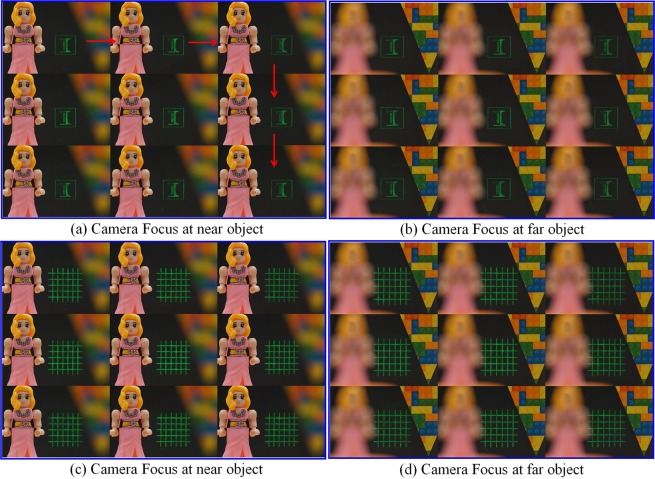
Images of test targets (“logo” and “grid”) seen through different pupils at varied focal distances. (**a**) Image of “logo” when camera focuses at a near object. (**b**) Image of “logo” when camera focuses at a far object (**c**) Image of “grid” when camera focuses at a near object. (**d**) Image of “grid” when camera focuses at a far object. See Supplementary Movies (Movies 1, 2, 3 and 4) for the dynamic focusing of camera and iris shifting.

Figure 6 shows the captured images of other two test images (an Illinois logo and a grid) seen from nine pupil locations when the camera focused at the near and far real-world objects. And we show the dynamic focusing process in supplementary movies (see Movies 1 and 2), where we fixed the iris at the upper-left pupil location and continuously varied its focus from the near object (2 diopters) to the far object (0.4 diopters). Due to the extension of the DOF of the eye imaging system by limiting the display beam width in converging propagation, the reconstructed target images remain in focus during focus adjustment while the two real-world objects appear sharp and blurred alternatively, proving the presentation of the always-focused images as expected. Other two supplementary movies (see Movies 3 and 4) record the process when we moved the iris at the pupil plane along the direction indicated by the red arrows in Fig. 6(a,c), visualizing the smooth image transition when iris changes between adjacent pupils. These results imply that we have created a Maxwellian-view image with an expanded eyebox in this optical see-through setup. And this is the first time such a system has been demonstrated in a lensless compact configuration—only a simple amplitude display is used.

## Discussions

### Light throughput

The light throughput of our system is mainly limited by both the display device and diffraction. First, in an amplitude display, each pixel modulates the light intensity by blocking the light transmission. For example, in our experiment, due to the use of a polarizer in front of the SLM, the light with only one polarization direction can pass. Similar to conventional amplitude displays, the light transmission at each pixel is determined by the voltage (or hologram gray-scale) response of SLM. Therefore, the light throughput varies pixelwise according to the displayed content. Additionally, for most liquid-crystal-based passive displays, the light experiences an additional loss due to the filling gap (fill factor) between pixels in either transmissive or reflective configuration. For example, the fill factor is 95.7% for the SLM used in our experiments. Although the light efficiency of amplitude displays is generally lower than that of their phase counterparts, the amplitude displays such as LCD and DLP are more accessible for consumer applications because of their low cost.

The second major light throughput loss is attributable to diffraction. Due to the pixelated structure of the amplitude displays, the light emitted from the display panel diffracts into different orders, each associated with a duplicated image. The common practice is to use only the zero-order diffraction (also known as SLM bandwidth) because it has the maximum energy (78% for the zero-order diffraction efficiency in our SLM). This efficiency can be improved by using an SLM with a smaller pixel.

### Reconstruction efficiency

The functionality of our method hinges on our ability to control both the phase and amplitude of the light wavefront to produce multiple converging beams carrying the image information. The encoding of a complex wavefront into an AO-CGH reduces the reconstruction efficiency because the AO-CGH contains the target complex wavefront as well as the DC and conjugated terms. The reconstruction efficiency can be numerically estimated through simulating the wavefront propagation from the encoded AO-CGH to the retina plane via pupil filtering. Also, to avoid the crosstalk between the signals and DC term, the incident beam on the hologram must have a slightly converging wavefront to increase the diffraction angle of the SLM.

To calculate the reconstruction efficiency, we first set all pixel amplitude values of the AO-CGH to unity and calculated the total power, *P*_0_, of the reconstructed image with no pupil filtering. Next, we computed the power *P*_1_ of the reconstructed image with an encoded AO-CGH and pupil filtering. We define the reconstruction efficiency as *P*_1_/*P*_0_, and this ratio largely depends on the image content. For example, the calculated reconstruction efficiency is ~0.2% for the “letters” image (Fig. 4a), ~0.1% for the “logo” image (Fig. 4b), and ~0.7% for the “grid” image (Fig. 4c).

### Eyebox size and field of view

We define the eyebox size as the area within which the Maxwellian-view image can be seen by the eye. In our proof-of-concept experiments, we form a 3 × 3 pupil array, and the distance between adjacent pupils is 1 *mm*. Therefore, the eyebox size is 3 *mm* × 3 *mm*. In general, when the eye pupil size is larger than the pupil spacing in pupil array, aliasing appears in the observed image where multiple duplicated Maxwellian-view images from two or more pupils overlap. In order to avoid image aliasing, the pupil distance must be greater than the physical eye pupil size, which varies from 1.5 *mm* to 8 *mm* dependent on the lighting condition. One possible solution is to update the AO-CGH by adjusting the plane carrier wave in Eq. (3) according to the detected eye pupil position and size from the pupil tracking device. The 1 *mm* pupil spacing in our experiments is limited by the small diffraction angle of the SLM, and this value can be increased for a smaller SLM pixel size.

**Figure 7:**
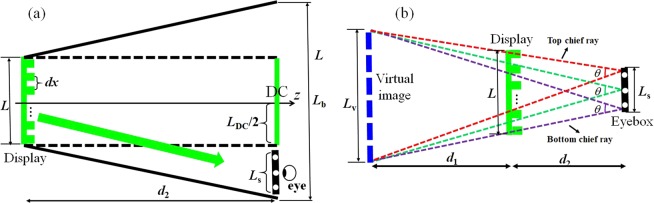
Calculation of (**a**) eyebox and (**b**) field of view.

In Fig. 7(a), we developed a theoretical framework to calculate the eyebox size. For simplicity, we used a one-dimensional model. Herein we denote the resolution and pixel pitch of the display as *N* and *dx*. The effective area of the display (AO-CGH) can be computed as *L* = *Ndx*, which is also the dimension of the DC term (*L*_DC_ = *L* = *Ndx*). Provided that the desired signals (i.e., pupil array), DC, and conjugated terms occupy the full bandwidth of the zero-order diffraction (*L*_b_ = *λd*_2_/*dx* under paraxial approximation^31^), to separate the off-axial signals from the DC term, the dimension of signal area *L*_s_ (i.e., eye box) must be no greater than *L*_b_/2- *L*_DC_/2, i.e.,

6 $$2{L}_{s}\le \frac{\lambda {d}_{2}}{dx}-Ndx.$$

To increase the eyebox area, Eq. 6 implies that we can increase the distance (*d*_2_), decrease the resolution (*N*), or reduce the pixel size (*dx*). However, for near-eye displays, a small *d*_2_ and a large *N* are desired because they yield a compact form factor and a high resolution, respectively. Therefore, the practical approach is to use a small pixel size. For example, to achieve 3 *mm* pupil spacing in a 9 × 9 pupil array, i.e., *L*_s_ = 3 *mm* × 3 = 9 *mm*, the required *dx* is 3.7 μm in our current setup.

We calculated the field of view (FOV) of our system based on geometrical optics. As shown in Fig. 7(b), for each focal spot in the effective eyebox, the chief rays emitted from the virtual image (*L*_v_) converge at the eye pupil via the display. The angle *θ* between the chief rays associated with the top and bottom of the virtual image defines the FOV. We assume this angle is approximately the same for all Maxwellian views seen from different pupils, and we calculate it as *θ* ≈ *L*_v_/(*d*_1_ + *d*_2_).

The FOV depends on the virtual image dimension *L*_v_, and it reaches the maximum when the chief rays associated with top and bottom pupil locations intercept the display screen edges as marked in Fig. 7(b). The maximum *L*_v-max_ and the correspondent FOV *θ*_max_ can be derived based on the trapezoidal geometry as:

7 $${L}_{v-\max }=\frac{L({d}_{1}+{d}_{2})-{L}_{s}{d}_{1}}{{d}_{2}},\,\,{\theta }_{\max }\approx \frac{{L}_{v-\max }}{({d}_{1}+{d}_{2})}=\frac{L}{{d}_{2}}-\frac{{L}_{s}{d}_{1}}{{d}_{2}({d}_{1}+{d}_{2})}.$$

In our proof-of-concept experiments, the size of virtual image *L*_v_ = 24 *mm*. The FOV is calculated as *θ* ≈ *L*_v_/(*d*_1_ + *d*_2_) = 24 *mm*/500 *mm* ≈ 2.75°, close to its maximum *θ*_max_ ≈ 2.8° defined by Eq. (7).

Equation 7 indicates that, given distances *d*_1_ and *d*_2_, there is a trade-off between the FOV *θ*_max_ and eyebox *L*_s_—increasing the FOV would unfavorably reduce the eyebox. To maintain the desired eyebox, we can alternatively reduce the distance *d*_2_. However, to display the correspondent hologram, the required pixel pitch becomes much smaller. For example, to increase the FOV by a factor of two, the required pixel pitch is 3.2μm compared to 9.2μm in the current setup. Alternatively, rather than using a plane illumination, we can shine a convergent wavefront onto the SLM to increase the FOV at the expense of using an additional lens^32^.

### Resolution and color reproduction

**Figure 8:**
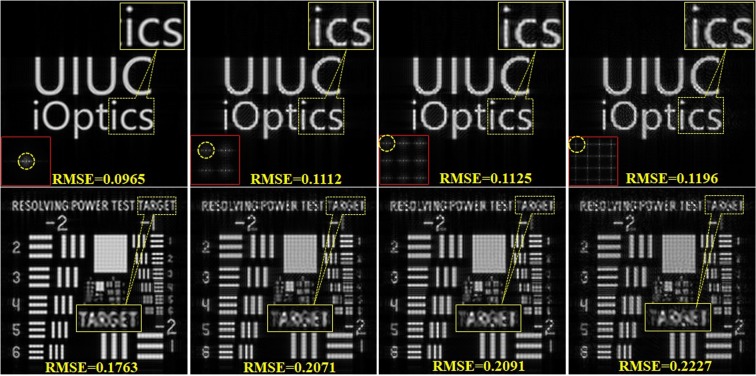
Evaluation of image quality under different pupil numbers.

The resolution of the AO-CGH in our experiment is 1024 × 1024 which is the same as the virtual target image. However, during the image reconstruction through diffraction, the high frequency information is lost due to pupil filtering. Also, the multiplexing of duplicated perspective views in to a single hologram reduces the information content of each Maxwellian-view image. To quantitatively evaluate the relations, we numerically reconstructed the Maxwellian-view image from one pupil view in different pupil array cases, and calculated the root mean square error (RMSE) values (all of the calculated intensities are normalized in [0, 1]) between each simulated reconstruction and the original target image. Figure 8 shows the simulation results for the two test images with the RMSE values marked in each image. Yellow dashed circles in each image column indicate the selected pupil positions of the numerical reconstructions from the pupil array of 1 × 1, 2 × 2, 3 × 3 and 4 × 4 cases. The reconstructions as well as the enlarged details imply that the quality and resolution of the image from single Maxwellian view degrade when the number of pupils increases, which is quantitatively verified by the RMSE values in each simulation result.

Although beyond the scope of current work, our method has an advantage in reproducing colors. Conventional HOE-based Maxwellian displays suffer from chromatic aberrations because the recorded interference pattern is wavelength dependent, causing both on- and off- axial miss-alignment of RGB channels in color mixing. Our method alleviates this problem because the modulation of light beams is achieved by a single AO-CGH. To reproduce colors, we can load three independent AO-CGHs into the RGB channels of the display and display them simultaneously. Then the RGB light emitted from these holograms propagates independently and merges at the retina, creating a color representation.

## Conclusions

In summary, we developed an optical see-through holographic Maxwellian near-eye display with an extended eyebox. We computationally generate an AO-CGH and display it on an amplitude-modulation device. The multiplexing of holograms on the AO-CGH enables pupil duplication, thereby significantly increasing the eyebox size. Because our system consists of only an amplitude display panel, it is simple and compact, lending an edge to its application in various wearable devices.

## Supplementary information

Supplementary Movies 1

Supplementary Movies 2

Supplementary Movies 3

Supplementary Movies 4
